# 14‐3‐3*ζ* binds to hepatitis B virus protein X and maintains its protein stability in hepatocellular carcinoma cells

**DOI:** 10.1002/cam4.1512

**Published:** 2018-10-24

**Authors:** Yufu Tang, Yibing Zhang, Chunhui Wang, Zhongyi Sun, Longfei Li, Jiahong Dong, Wenping Zhou

**Affiliations:** ^1^ Department of Hepatobiliary Surgery The General Hospital of Shenyang Military Area Command Shenyang 100016 China; ^2^ Post‐doctoral Station The General Hospital of Shenyang Military Area Command Shenyang 10016 China; ^3^ Department of Hepatobiliary and Pancreas Surgery Beijing Tsinghua Changgung Hospital (BTCH) School of Clinical Medicine Tsinghua University Beijing China

**Keywords:** 14‐3‐3*ζ*, Akt signaling pathway, hepatitis B virus protein X, hepatocellular carcinoma, portal vein tumor thrombosis

## Abstract

14‐3‐3*ζ*, a phosphopeptide‐binding molecule, is reportedly overexpressed in the cancerous tissues of patients with hepatocellular carcinoma (HCC). Hepatitis B virus (HBV) protein X (HBx) draws intensive attention in HBV‐related HCC because it not only regulates HBV replication, but also promotes carcinogenesis by interacting with various tumor or antitumor molecules. This study is performed to investigate whether and how 14‐3‐3*ζ* interacts with HBx. The coimmunoprecipitation (Co‐IP) results showed that 14‐3‐3*ζ* bond to HBx in HBV‐infected Hep3B HCC cells and CSQT‐2 portal vein tumor thrombosis (PVTT) cells. By performing Co‐IP assay in HBV‐free Huh7 cells expressing wild‐type HBx, mutant HBx‐S31A, or HBx‐S31D (serine^31^ was mutated into alanine^31^ or aspartic acid^31^), we found that the phosphorylated serine^31^ with its near amino acid residues constituted a RPLphosphoS^31^ GP (R, arginine; P, proline; L, leucine; S, serine; G, glycine) motif in HBx for 14‐3‐3*ζ* docking. This 14‐3‐3*ζ*‐HBx interaction was partly impaired when Akt signaling transduction was blocked by LY294002. Furthermore, 14‐3‐3*ζ* silencing augmented HBx ubiquitination and decreased its expression in cancer cells and xenograft tumor. The migratory and invasive abilities of CSQT‐2 cells were inhibited upon 14‐3‐3*ζ* silencing, whereas partly restored by HBx overexpression. Additionally, 14‐3‐3*ζ* positively correlated with HBx to be overexpressed in the primary HCC tissues (*r* = 0.344) and metastatic PVTT (*r* = 0.348). In summary, findings of this study reveal a novel 14‐3‐3*ζ*‐HBx interaction in HCC cells and suggest 14‐3‐3*ζ* as a candidate target for treating HBV‐related HCC.

## Introduction

Primary liver cancer ranks the fifth leading cancer in men and the ninth in women, with 70–90% of new cases/year being hepatocellular carcinoma (HCC) 1. Hepatitis B virus (HBV) belongs to the *Hepadnaviridae* family and is a small DNA virus with partially double‐stranded genome 2, 3. Chronic infection with HBV is a well‐known cause of cirrhosis and liver cancer, particularly in Asian countries 4. Upon infection, HBV enters into a host cell to form covalently closed circular DNA (cccDNA) that exists as a minichromosome 2. Viral cccDNA from infected hepatocytes cannot be cleared because of the integration of HBV DNA into the host genome 5, 6.

14‐3‐3 proteins are phosphopeptide‐binding molecules which are involved in multiple cellular processes, including signaling transduction, cell‐cycle regulation, and cell apoptosis 7, 8. 14‐3‐3*ζ* is a member of 14‐3‐3 family, and its elevation has been reported in several malignant tumors, such as breast cancer 9 and lung cancer 10, and thus, it is considered as a promising therapeutic target in cancer. Several tumorigenesis‐related proteins are proven to interact with 14‐3‐3 members upon phosphorylation, such as E2F1 10 and F‐box protein 4 11. Choi et al. reported an aberrant overexpression of 14‐3‐3*ζ* in several HCC cell lines and in human hepatic tumor tissues 12. They also demonstrated that HCC cells with decreased 14‐3‐3*ζ* displayed impaired proliferation and tumorigenicity. These previous findings suggest a protumor function of 14‐3‐3*ζ* in liver cancer 12. Portal vein tumor thrombosis (PVTT) arising from the invasion of cancer cells into the portal vein is considered as a major complication that links to poor survival 13. However, whether 14‐3‐3*ζ* is associated with this special metastatic type of HCC is unclear.

Hepatitis B virus genome contains four overlapping open reading frames (ORFs) that encode seven proteins 3. The intact HBV virus X protein (HBx) is encoded by the smallest viral ORF and is a 17 kDa protein with 154 amino acids 3. Increasing attention has been drawn to HBx, not only because of its essential role in viral replication, but also because of its involvement in cell‐cycle regulation and DNA repair during liver chronic necroinflammation 14. HBx contributes to the tumorigenesis of HCC by interacting with protumor and/or antitumor molecules 15, 16. Of note, it also promotes the metastasis of HCC by regulating molecules associated with the migration and invasion of tumor cells, such as membrane‐type matrix metalloproteinase‐1, cyclooxygenase‐2, and beta 1 integrins 17, 18. The identification of novel molecules that disturb the expression of HBx will provide insights into HCC therapy. Two different binding motifs (RXXpSXP, RXXXpSXP) present in nearly all known 14‐3‐3‐binding proteins, where R represents arginine, S or pS represents serine or phospho‐serine, P represents proline, and X represents any amino acid 7. Interestingly, Lee and coworkers demonstrated that HBX could be phosphorylated at serine^31^ by Akt 19. This phospho‐serine^31^ of HBx generates a potential docking site for 14‐3‐3s. Herein, we propose that 14‐3‐3*ζ* plays a role in HBV‐related HCC by interacting with HBx.

In this study, we first investigated the interaction between 14‐3‐3*ζ* and HBx in two HCC cell lines, Hep 3B and Huh7 cells, and in a PVTT cell line, CSQT‐2 cells 20. We found that 14‐3‐3*ζ* bond to HBx in HBV‐positive Hep 3B and CSQT‐2 cells. 14‐3‐3*ζ*‐silenced cancer cells displayed weakened migration and invasion, which was accompanied by decreased HBx expression.

## Materials and Methods

### Patients

Eighteen patients diagnosed with HCC/PVTT in the General Hospital of Shenyang Military Area Command were enrolled. No neoadjuvant therapy was applied in these patients before sampling. Eighteen sets of tissue samples (nontumor adjacent tissue, primary tumor, and PVTT) were collected between October 2011 and July 2012 and stored appropriately until use. This work was carried out in accordance with the Declaration of Helsinki, and a written informed consent has been obtained from each participant.

### Cell culture

Hepatitis B virus‐infected cell line Hep3B 21 and noninfected cell line Huh7 22 were obtained from Shanghai Zhong Qiao Xin Zhou Biotechnology Co.,Ltd. (Shanghai, China) and used in the present experiments within 6 months after receipt. A cell line CSQT‐2 (HBV‐infected) with high metastatic potential was kindly provided by Prof. Shuqun Cheng (Eastern Hepatobiliary Surgery Hospital, Second Military Medical University) and used as an in vitro model for PVTT. Cells were cultured in DMEM (Gibco, Grand Island, NY) with 10% fetal bovine serum (HyClone, Logan, UT) in a humidified 5% CO_2_/95% air environment at 37°C.

### Vector construction, cell transfection, and viral infection

Sequence encoding the whole wild‐type HBx (HBx‐wt) of HBV adw subtype 23 was synthesized by Sangon Biotech (Shanghai, China) and was inserted into the pFLAG‐CMV expression vector. HBx‐S31A or HBx‐S31D mutant was generated by specific primers that replaced TCG (a triplet codon of serine) with GCT (a triplet codon of alanine) or GAT (a triplet codon of aspartic acid) and then was inserted into the expression vector. Then, these plasmids were encapsulated with Lipofectamine 2000 (Invitrogen, Carlsbad, CA) to transfect the Huh7 cells according to the manufactory’s protocols.

14‐3‐3*ζ* shRNA and the nontargeting shRNA were synthesized and inserted into pGLVH1/GFP+Puro lentiviral vector (GenePharma Co., Ltd, Shanghai, China), and the lentiviral particles were generated in 293T packaging cells according to the manufactory’s instructions. Hep3B cells and CSQT‐2 cells were infected with lentiviral particles and cultured in complete DMEM containing puromycin (Santa Cruz Biotechnology, Santa Cruz, CA) to select the 14‐3‐3*ζ*‐silenced cell clones.

### Quantitative real‐time PCR

The quantitative real‐time PCR was carried out to detect the mRNA expression of 14‐3‐3*ζ* in tissue specimens. In short, the total RNAs were extracted by using a RNApure fast extraction kit (BioTeke China, Beijing, China), and the cDNA library was obtained by using a transcriptor first strand cDNA synthesis Super M‐MLV kit (BioTeke China). A pair of qRT‐PCR primers for *14‐3‐3ζ* gene was designed: forward, 5′ GCCATTGCTGAACTTGATA 3′; reverse: 5′ GCTTCGTCTCCTTGGGTAT 3′. The mRNA expression of 14‐3‐3*ζ* was calculated based on 2^−Δct^ against the control *β*‐actin 24.

### Immunohistochemical staining

Slices of tissue samples were deparaffinized in xylene, dehydrated in graded alcohol series, and washed with distilled water before subjecting to antigen retrieval in the boiling citrate buffer. Then, these slices were rinsed with PBS, incubated with 3% H_2_O_2_ at room temperature (RT) for 15 min, and blocked with goat serum at RT for 15 min. Mouse anti‐HBx monoclonal antibody (1:200; Abcam, Cambridge, MA) or rabbit anti‐14‐3‐3*ζ* polyclonal antibody (1:200; Abcam) was used to incubate tissue slices at 4°C overnight. Thereafter, these sections were incubated with biotin‐labeled goat anti‐rabbit or anti‐mouse secondary antibody (1:200; Beyotime, Shanghai, China) at 37°C for 30 min, and then with HRP‐labeled streptavidin at 37°C for additional 30 min. The expression signal was magnified by 100 *μ*L DAB, and the cell nuclei were counterstained with hematoxylin.

### Coimmunoprecipitation and western blotting assay

Total proteins were extracted for coimmunoprecipitation (co‐IP) assay. In brief, 400–500 *μ*g protein was incubated with 2 *μ*L anti‐14‐3‐3*ζ* at 4°C overnight, and then with 40–60 *μ*L Protein A+G Agarose for 2–3 h. This mixture was centrifuged, washed with PBS, and then resuspended in 40–60 *μ*L loading buffer (2×). After boiling for 5 min, 20–40 *μ*L sample was subjected to Western blotting assay. Equal volume of each protein sample was separated on a 10% SDS‐PAGE and transferred onto PVDF sheets. The PVDF membranes containing target protein were blocked with skim milk, and then incubated with anti‐HBx (1:1000; Abcam), antiflag (1:500; CW‐Biotech, Beijing, China), or anti‐Ubiquitin antibody (1: 500; ProteinTech, Rocky Hill, NJ) at 4°C overnight, and then with proper secondary antibody (1: 5000, Beyotime) at 37°C for 45 min. After washing using TBST, the protein blots were developed by using an ECL reagent, and the densities were analyzed by Gel‐Pro Analyzer software. For Western blotting, total protein samples were used in the assay, with *β*‐actin as an endogenous reference. The primary anti‐Akt^phospho S473^ (1:1000) and anti‐Akt (1:5000) antibodies were purchased from Abcam.

### Detection of cell migration and invasion

Wound healing assay was performed to determine cell migration. In short, cells grown into confluence were pretreated with 1 *μ*g/mL mitomycin C (Sigma, Sigma‐Aldrich, St. Louis, MO, USA) for 1 h, and then were scratched with a 200‐*μ*L sterile pipette tip on the cell monolayer. Cell images were taken under a light microscope in the beginning and at 24 h later. For transwell assay, matrigel (BD Biosciences, San Jose, CA)‐coated transwell chambers (Corning, Tewksbury, MA) were placed in a 24‐well plate containing DMEM plus 20% FBS. Cells (2 × 10^4^) in 200 *μ*L serum‐free DMEM were seeded into the upper chamber. Cells invaded through the chamber membrane were fixed in 4% paraformaldehyde at RT for 20 min and then stained with 0.5% hematoxylin.

### Xenograft model in nude mice

CSQT‐2 cells (1 × 10^7^ in 0.2 mL) were injected into mouse axillary skin. Tumor nodes appeared from day 10, and their volumes were measured every 3 days since then. All mice were sacrificed on day 34 to collect the tumor mass. The animal experiments complied with the Guide for the care and use of Laboratory animals and were approved by the Research Ethics Board of the General Hospital of Shenyang Military Area Command.

### Statistical analysis

All data were shown in the form of mean ± standard deviation (SD) and analyzed with SPSS version 20.0 (IBM, Armonk, NY). Paired student’s *t* test (two tailed) was applied to comparing the 14‐3‐3*ζ* expression in tissue samples between two groups, while unpaired student’s *t* test was performed to analyze data from two groups for the rest study. One‐Way ANOVA followed by Bonferroni’s multiple comparison test was performed to analyze data from ≥ three groups. A *P* value < 0.05 was considered significant. Expression fold changes of 14‐3‐3*ζ* and HBx were calculated by comparing their protein expression levels in primary tumor and PVTT tissues to that in nontumor tissue and then analyzed with Pearson correlation.

## Results

### 14‐3‐3*ζ* interacts with p^Ser31^‐HBx in HCC and PVTT cells in vitro

**Figure 1:**
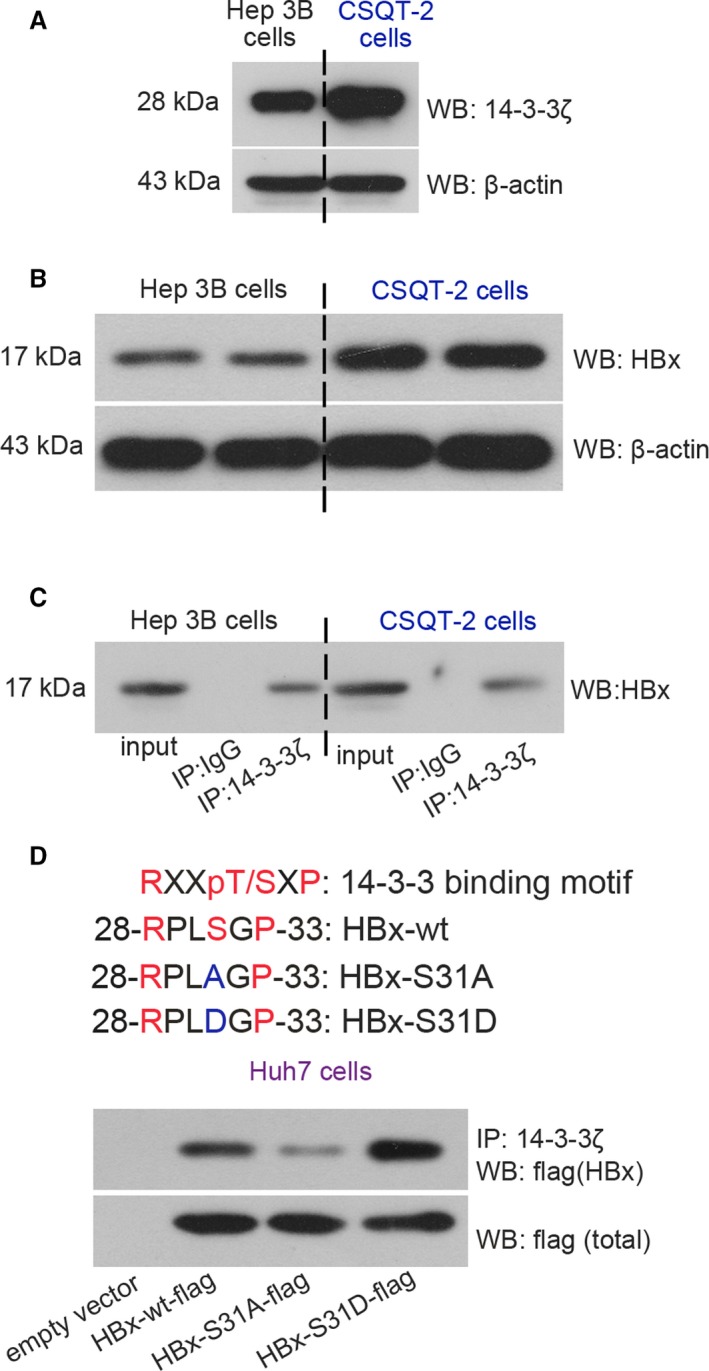
14‐3‐3*ζ* binds to pSer31‐HBx in HCC cells in vitro. The basal expression levels of (A) 14‐3‐3*ζ* and (B) HBx in Hep 3B and CSQT‐2 cells were determined with Western blotting analysis. (C) Co‐IP assay was performed to detect 14‐3‐3*ζ*‐HBx interaction in Hep 3B and CSQT‐2 cells. (D) Huh7 cells were transfected with empty vector, HBx‐wt, HBx‐S31A, or HBx‐S31D mutant plasmid, and the transfection efficiency was determined with Western blotting using antiflag antibody. Co‐IP assay was performed to detect 14‐3‐3*ζ*‐HBx interaction in Huh7 cells. R, arginine; P, proline; L, leucine; S, serine; G, glycine; D, aspartic acid; wt, wild type.

Results from Western blotting analysis illustrated that Hep3B cells and CSQT‐2 cells were 14‐3‐3*ζ*‐ and HBx‐positive cells (Fig. 1A,B). Both Hep 3B and CSQT‐2 cells expressed a full‐length HBx protein (17 kDa) (Fig. 1B). The subsequent co‐IP assay demonstrated a novel 14‐3‐3*ζ*‐HBx coexistence in Hep3B cells and CSQT‐2 cells (Fig. 1C). By analyzing the amino acid sequence of HBx of Hep3B cells 25, we found that the phosphorylation of HBx on serine^31^ may provide a recognition site for 14‐3‐3s. Thus, Huh7 cells (a HBV‐free cell line) transfected with HBx‐wt or HBx‐S31A (serine^31^ was mutated into alanine^31^) plasmid were subjected to co‐IP and Western blotting assays. The expression of flag‐tagged HBx could be probed in HBx‐wt, HBx‐S31A or HBx‐S31D (phospho‐mimic mutant) plasmid‐transfected Huh7 cells, but not in empty vector‐transfected cells (Fig. 1D). Interestingly, our data showed that the binding of 14‐3‐3*ζ* and HBx was suppressed when serine^31^ on HBx was mutated into alanine^31^, whereas augmented when it mutated into aspartate^31^ (Fig. 1D). Collectively, these results revealed the colocalization of 14‐3‐3*ζ* and HBx in HCC cells, and the RPLphosphoS^31^GP (R, arginine; P, proline; L, leucine; S, serine; G, glycine) domain of HBx was a docking site for 14‐3‐3*ζ* binding.

### Activated Akt contributes the interaction between 14‐3‐3*ζ* and pSer^31^‐HBx

**Figure 2:**
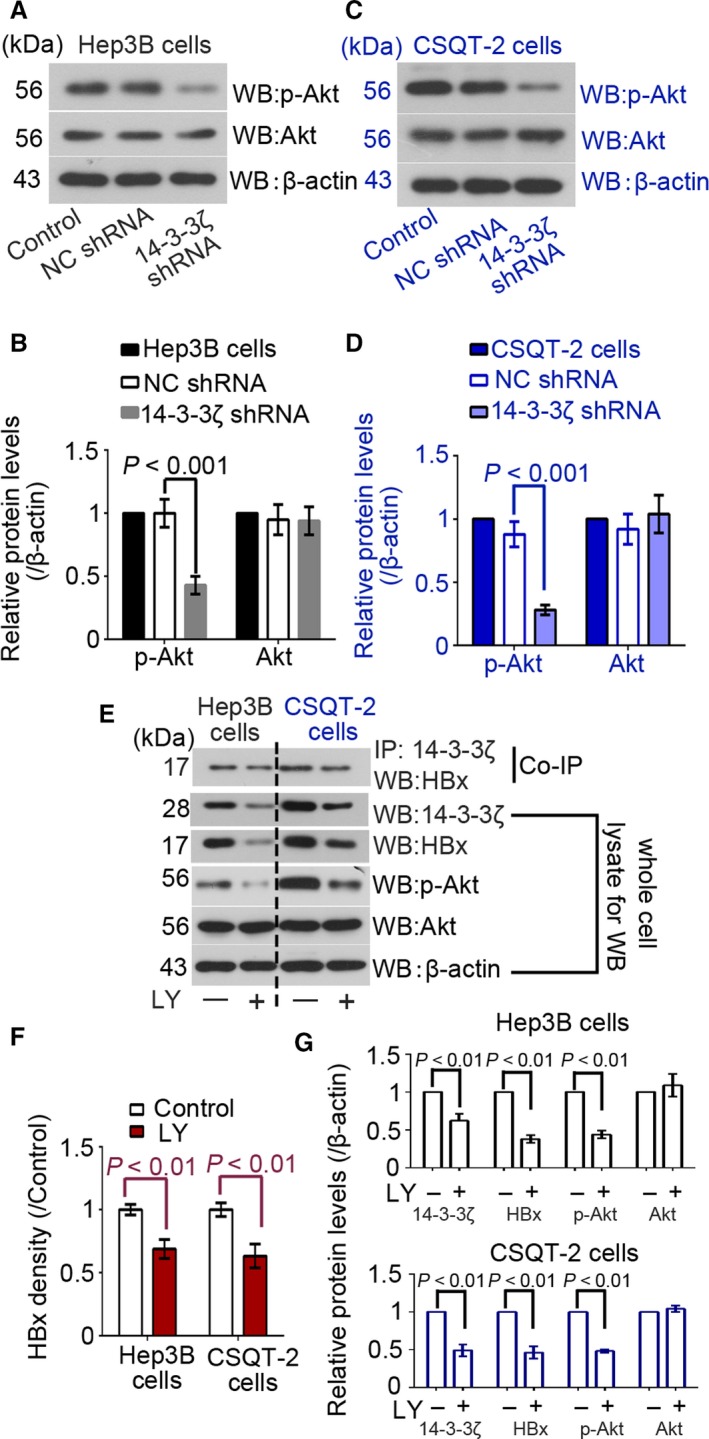
Inhibition of Akt pathway suppresses the interaction between 14‐3‐3*ζ* and pSer^31^‐HBx. Hep 3B and CSQT‐2 cells were infected with lentiviral particles containing 14‐3‐3*ζ* shRNA or NC shRNA. The levels of p‐Akt and total Akt in Hep 3B (A and B) and CSQT‐2 cells (C and D) were determined with Western blotting analysis with *β*‐actin as an endogenous reference (*n* = 3). Hep 3B and CSQT‐2 cells were treated with or without LY294002 (20 *μ*mol/L) for 24 h. (E and F) IP with anti‐14‐3‐3*ζ* antibody and Western blotting assay with anti‐HBx antibody were performed in Hep 3B and CSQT‐2 cells. (E and G) The levels of 14‐3‐3*ζ*, HBx and phosphorylated‐Akt (p‐Akt) and total Akt were determined with Western blotting analysis with *β*‐actin as an endogenous reference (*n* = 3).

We next investigated whether Akt pathway was involved in the interaction between 14‐3‐3*ζ* and HBx. Results of Western blotting analysis indicated that the phosphorylation of Akt both in Hep3B and CSQT‐2 cells was inhibited upon 14‐3‐3*ζ* knockdown (Fig. 2A–D). Furthermore, LY294002 was used to block Akt signaling transduction in these cells to determine whether the activation of Akt signaling pathway contributed to 14‐3‐3*ζ*‐HBx binding. Additional co‐IP results showed that the 14‐3‐3*ζ*‐HBx binding in these two cancer cell lines was partly impaired by LY294002 (Fig. 2E,F). The expression of 14‐3‐3*ζ* and HBx was downregulated by LY294002 as well (Fig. 2E,G). These findings indicated that the inhibition of Akt pathway suppressed the binding between 14‐3‐3*ζ* and HBx, and that 14‐3‐3*ζ* itself affected the activation of Akt pathway.

### Knockdown of 14‐3‐3*ζ* inhibits HBx expression

**Figure 3:**
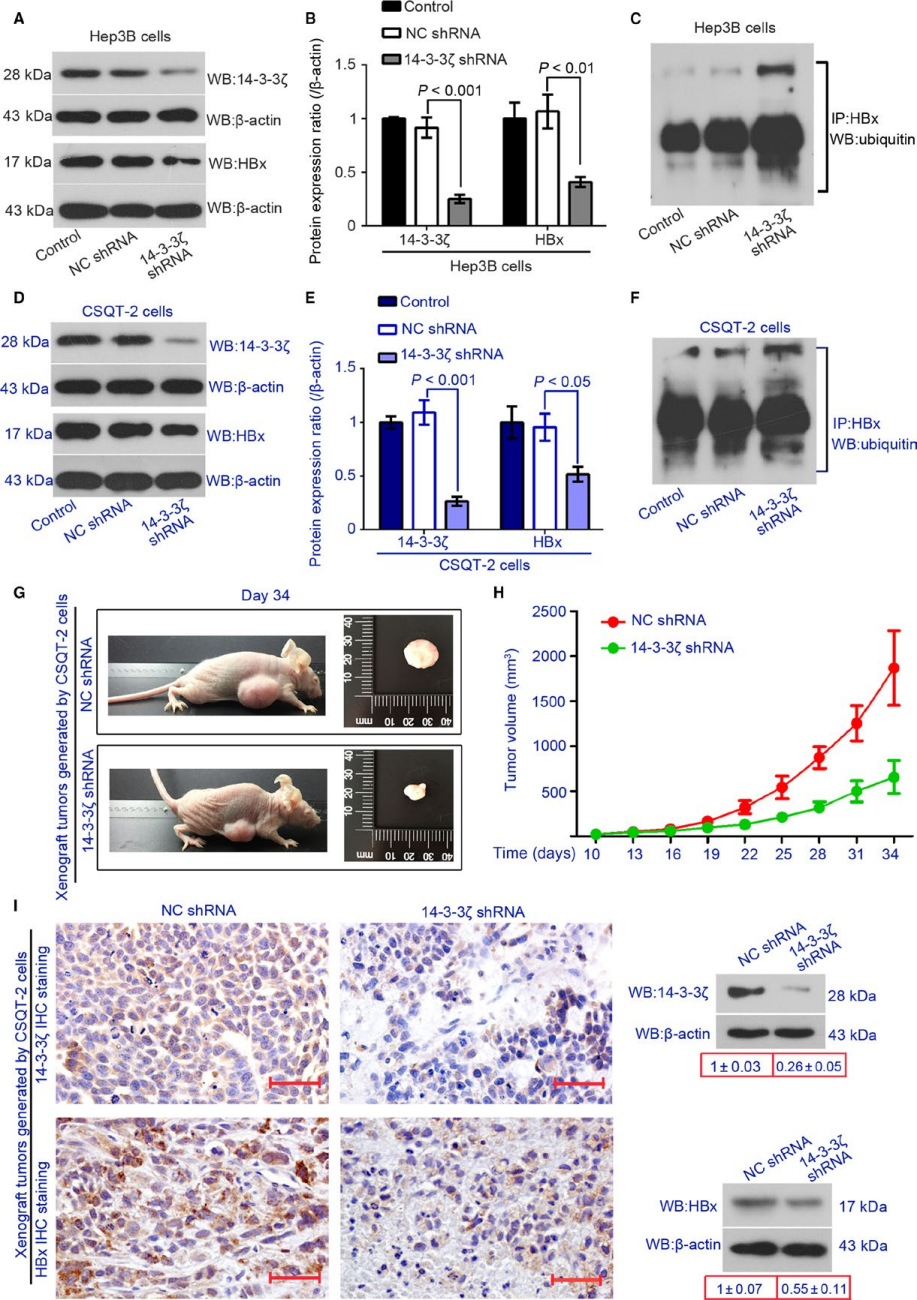
Knockdown of 14‐3‐3*ζ* inhibits HBx expression in vitro and in vivo and induces its ubiquitin‐mediated degradation. Cells were infected with lentiviral particles containing 14‐3‐3*ζ* shRNA or NC shRNA. The expression levels of 14‐3‐3*ζ* and HBx in Hep 3B (A and B) and CSQT‐2 cells (D and E) were determined with Western blotting analysis with *β*‐actin as an endogenous reference (*n* = 3). IP with anti‐HBx antibody and Western blotting assay with anti‐Ubiquitin antibody were performed in Hep 3B (C) and CSQT‐2 cells (F). (G) Xenograft tumors generated by CSQT‐2 cells expressing NC shRNA or 14‐3‐3*ζ* shRNA were photographed, and (H) their volumes were calculated every 3 days since day 10 after cell injection (*n* = 6). (I) 14‐3‐3*ζ* and HBx protein expression levels were analyzed in xenograft tumors via IHC staining (bars = 50 *μ*m; right) and Western blotting analysis (left).

Lentiviral particles containing 14‐3‐3*ζ* shRNA or NC shRNA were used to establish 14‐3‐3*ζ*‐silenced Hep3B and CSQT‐2 cells. The knockdown efficiency of 14‐3‐3*ζ* shRNA was confirmed by performing Western blotting using anti‐14‐3‐3*ζ* antibody. We found that 14‐3‐3*ζ* shRNA decreased the protein expression 14‐3‐3*ζ* by 73% in Hep3B cells (Fig. 3A,B) and by 76% in CSQT‐2 cells (Fig. 3D,E). HBx expression was downregulated in 14‐3‐3*ζ*‐silenced cells as compared to the control cells (Fig. 3). We next investigated the ubiquitination of HBx in Hep3B and CSQT‐2 cells. IP with anti‐HBx antibody and Western blotting with anti‐Ubiquitin antibody were performed. We found that the silencing of 14‐3‐3*ζ* increased ubiquitinated HBx in both cell lines (Fig. 3C,F). In addition, the xenograft tumors formed by 14‐3‐3*ζ*‐silenced CSQT‐2 cells were much smaller than that formed by control CSQT‐2 cells (Fig. 3G,H). Like in vitro, HBx expression decreased in the xenograft tumors when 14‐3‐3*ζ* was silenced (Fig. 3I).

### 14‐3‐3*ζ* shRNA reduces the migration and invasion of CSQT‐2 cells in vitro

**Figure 4:**
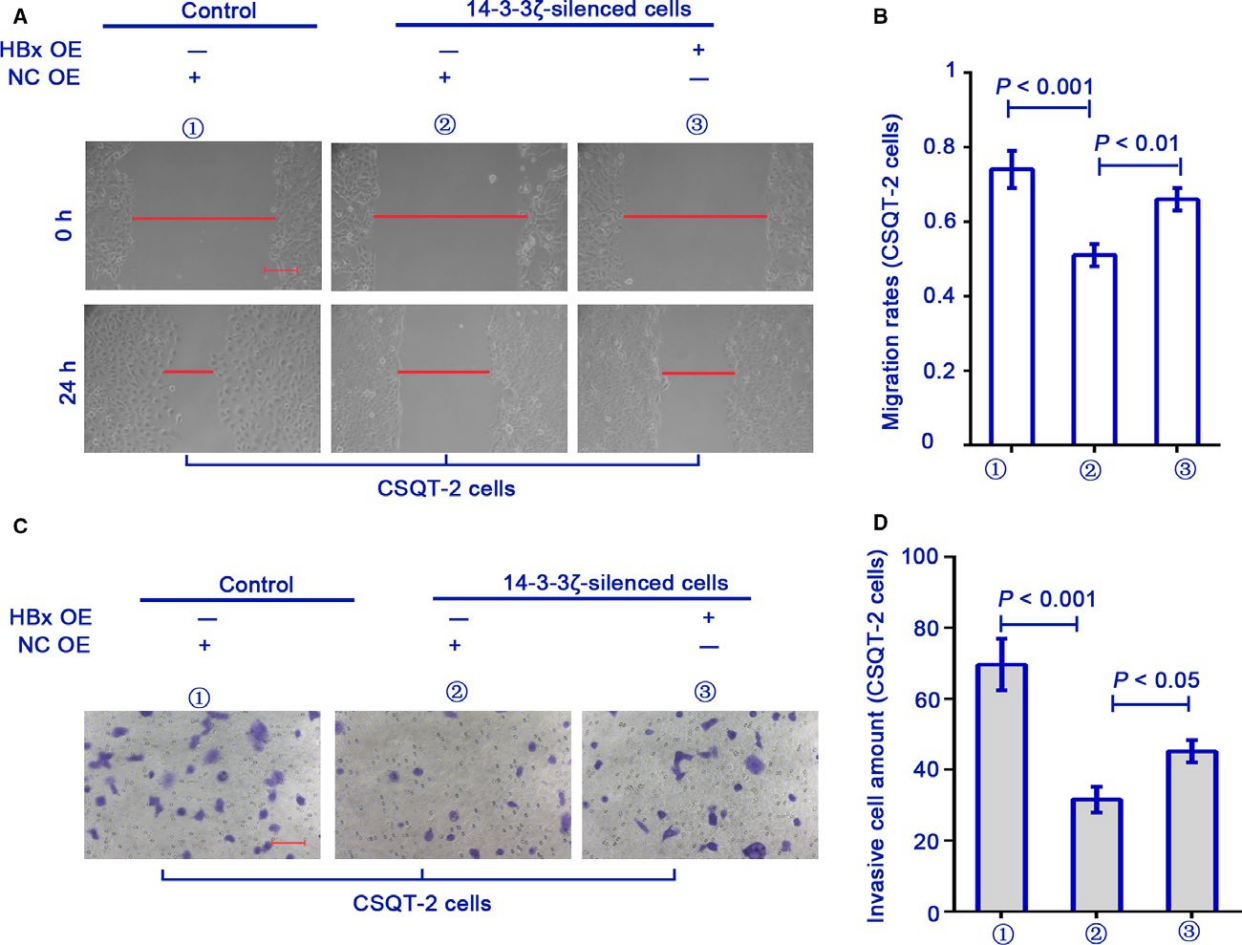
14‐3‐3*ζ* shRNA reduces migration and invasion of CSQT‐2 cells. Control (NC shRNA) or 14‐3‐3*ζ*‐silenced CSQT‐2 cells were further transfected with empty vector or HBx‐wt plasmid. (A and B) The migration and (C and D) invasion of CSQT‐2 cells were detected with wound healing assay and matrigel‐coated transwell assay, respectively. Bars in part A = 200 *μ*m. Bars in part C = 100 *μ*m. Data were expressed as mean ± SD (*n* = 3).

Furthermore, the migration and invasion of control and 14‐3‐3*ζ*‐silenced CSQT‐2 cells were determined with scratch assay and matrigel‐coated transwell assay, respectively. We found that the mobility of 14‐3‐3*ζ*‐silenced CSQT‐2 cells was weaker than the control cells (Fig. 4A–D). The decreased migration and invasion of 14‐3‐3*ζ*‐silenced cells were partly rescued if HBx was reexpressed (Fig. 4A–D). These results suggested that HBx was involved in 14‐3‐3*ζ*‐mediated invasiveness of PVTT cells in vitro.

### 14‐3‐3*ζ* correlates with HBx to be overexpressed in primary and metastatic HCC tissues

**Figure 5:**
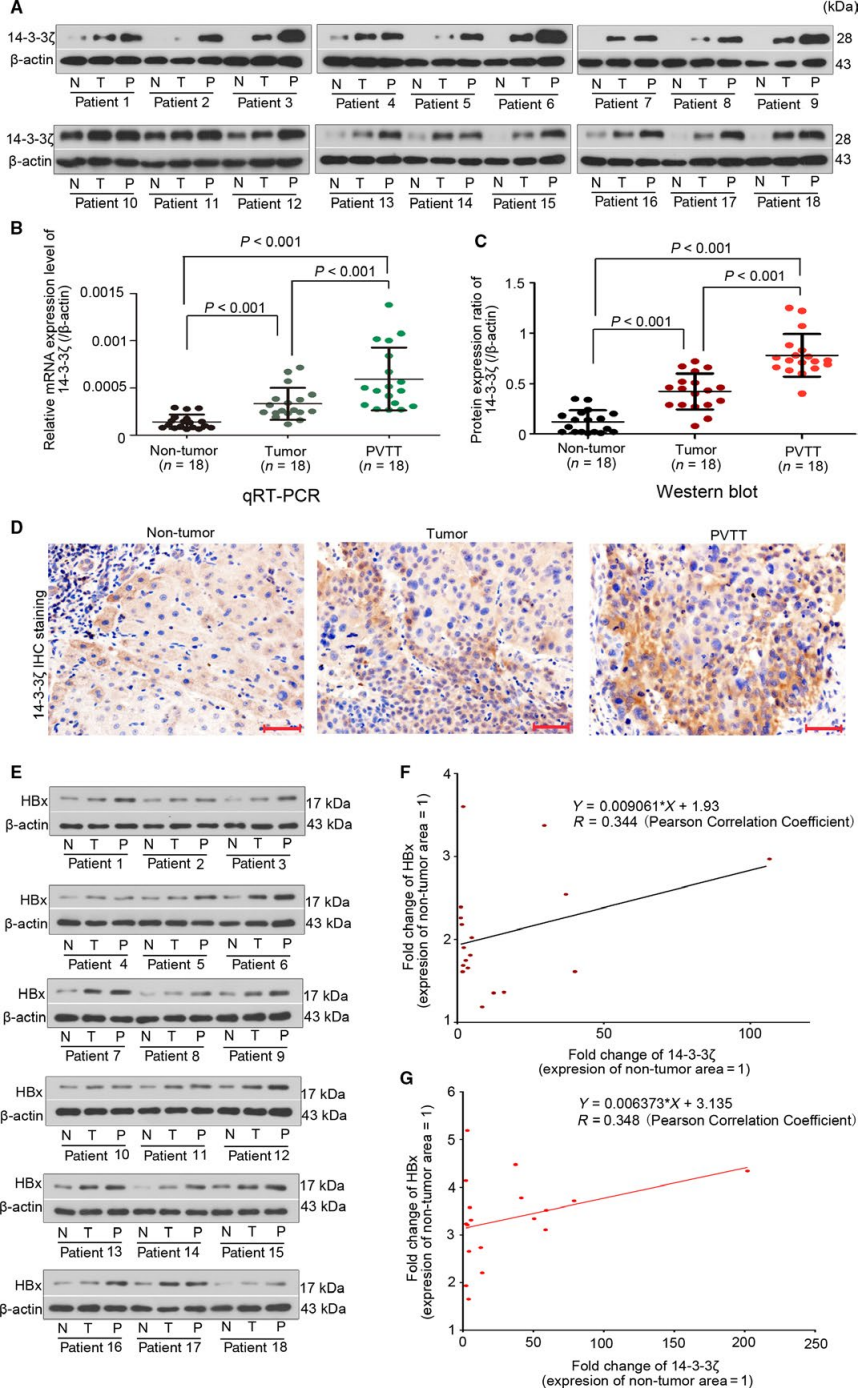
14‐3‐3*ζ* correlates with HBx to be elevated in primary and metastatic tumor tissue of HCC patients. The 14‐3‐3*ζ* protein expression in nontumor, primary tumor, and PVTT tissue (*n* = 18 pairs) was analyzed with (A,C) Western blotting analysis (with *β*‐actin as an endogenous reference) and (D) IHC staining (Bars = 50 *μ*m). (B) Its mRNA expression was determined with qRT‐PCR analysis. The protein levels of HBx were also determined via Western blotting analysis with *β*‐actin as an endogenous reference (E). Fold changes of 14‐3‐3*ζ* and HBx were calculated by comparing their protein expression levels of the primary tumor (F) and PVTT tissues (G) to that of the nontumor tissue and then analyzed with Pearson correlation.

Finally, we assessed the expression of 14‐3‐3*ζ* in HCC specimens by carrying out qRT‐PCR, Western blotting, and Immunohistochemical (IHC) staining assay. The mRNA level of 14‐3‐3*ζ* in primary tumor and PVTT tissue was approximately twofold and fourfold as compared to that in peri‐tumor tissue (Fig. 5B). 14‐3‐3*ζ* protein expression showed a similar expression pattern in these samples (Fig. 5A,C). Results from IHC assay showed that the intensity of 14‐3‐3*ζ* was PVTT > primary tumor > peri‐tumor area (Fig. 5D). Furthermore, the protein expression of HBx was also determined with Western blotting assay (Fig. 5E). Fold change (FC) of 14‐3‐3*ζ* or HBx was calculated by comparing their protein expression levels of primary tumor and PVTT tissues to that of nontumor tissue. Results from Pearson correlation analysis showed that the FC of HBx was positively correlated with that of 14‐3‐3*ζ* in primary tumor (Fig. 5F, *r* = 0.344) and PVTT (Fig. 5G, *r* = 0.348).

## Discussion

14‐3‐3 family members are reported to be overexpressed in a diverse array of tumors: 14‐3‐3*β* in gastric cancer 26, 14‐3‐3*σ* in pancreatic ductal adenocarcinoma 27, and 14‐3‐3*τ* in breast cancer 28. 14‐3‐3*ζ* is initially identified in 1996 by Seluja et al. 29, and like the other 14‐3‐3 members, it is elevated in the cancerous area of various malignancies and contributes to the carcinogenesis. For instance, 14‐3‐3*ζ* was identified as an androgen‐responsive factor, and its high expression was linked to malignancy and lymph node metastasis in prostate cancer 30. 14‐3‐3*ζ* could turn the role of transforming growth factor *β* from a tumor suppressor to a metastatic driver in breast cancer 9. Our group prior demonstrated that 14‐3‐3*ζ* contributed HCC carcinogenesis by interacting with the oncogenic factor hypoxia‐inducible factor‐1*α* 31. HBV infection is the predominant cause of HCC in Asian countries, especially in China 4. 14‐3‐3*ζ* could interact with and maintain the expression of human papillomavirus (HPV) E6 oncoprotein 32, implying an involvement 14‐3‐3*ζ* in human virus‐related carcinogenesis. We thus carried out the present work to explore the role of 14‐3‐3*ζ* in HBV‐related HCC, with focuses on HBx.

By analyzing the amino acid sequence of HBx, we found a potential binding site for 14‐3‐3s within HBx, RPLphosphoS^31^GP domain. Akt can induce phosphorylation of HBx on at serine^31^ 19, which may provide a potential docking site for 14‐3‐3s. By transfecting the Huh7 cells with HBx S31A or S31D plasmid, we confirmed that the phosphorylation of HBx on at serine^31^ was responsible for the bond of HBx to 14‐3‐3*ζ*. Both HBx S31A and S31D plasmids are resulted from missense mutation. It is possible that not only the phosphorylation status of HBx is changed, the structure and biological function of HBx may also be unexpectedly altered. In this regard, our group chose to investigate how 14‐3‐3*ζ* affected HBx expression in HCC cells expressing the full‐length of wild‐type HBx protein (17 kDa), Hep 3B, and CSQT‐2 cells.

HBx has long been considered as a contributor for HBV‐infected HCC development 33, 34, 35. Diao et al. have demonstrated that 14‐3‐3*β*, another member of 14‐3‐3 family, can bind to HBx by recognizing the phosphorylated serine^31^ 36. However, they did not elucidate whether and how this 14‐3‐3 subtype mediated HBx expression. It has been reported that HBx can be degraded in an ubiquitination‐dependent way 37, 38, and that the 14‐3‐3 members can affect their binding partners’ stabilization by promoting or inhibiting the ubiquitination 39, 40. Given the fact that the expression of HBx was downregulated when 14‐3‐3*ζ* was inhibited by its shRNA, we proposed that the formation of 14‐3‐3*ζ*‐HBx complex may prevent HBx from ubiquitin‐mediated degradation. Data showing that the level of ubiquitinated HBx was upregulated in 14‐3‐3*ζ*‐silenced HCC cells verified our hypothesis. The formation of 14‐3‐3*ζ*‐HBx complex is hindered because of the 14‐3‐3*ζ* knockdown, leading to the reduction in HBx. It is plausible that 14‐3‐3*ζ* is responsible for maintaining the intracellular expression of HBx.

Our data further illustrated that the interaction between 14‐3‐3*ζ* and HBx was partly impaired when Akt signaling transduction was suppressed by LY294002, implying that the activated Akt signaling‐mediated HBx phosphorylation contributed to 14‐3‐3*ζ*‐HBx binding. A recent study demonstrated that 14‐3‐3*ζ* loss induced deactivation of Akt pathway in mammary tumors 41. Our work in line with this earlier study revealed that 14‐3‐3*ζ* silencing suppressed Akt signaling transduction in HCC cells. The blockade of Akt pathway induces the dephosphorylation of HBx, leading to a weakened interaction between HBx and 14‐3‐3*ζ*. If this pathway is suppressed, the expression of HBx is anticipated to be downregulated. As expected, HBx was downregulated in both Hep 3B cells and CSQT‐2 cells treated with LY294002. Interestingly, the expression level of 14‐3‐3*ζ* was also decreased. These data support the existence of a positive feedback loop between 14‐3‐3*ζ*/HBx and Akt pathway. Further experiments are needed to elucidate the underlying mechanisms of this feedback loop.

In addition, as the two used cell lines were 14‐3‐3*ζ* positive cells, we only performed loss‐of‐function experiments of 14‐3‐3*ζ* in these cells. To confirm the binding of 14‐3‐3*ζ* to HBx can augment the protein stabilization of HBx, further gain‐of‐function study will be performed in 14‐3‐3*ζ* negative HCC cells by our team.

The p38 mitogen‐activated protein kinase (MAPK)‐activated protein kinase‐2 (MK2) was observed to augment the binding between 14‐3‐3*ε* and 14‐3‐3*ζ* with CEP131 by phosphorylating CEP131 on serine 47 and 78 42. In fact, we also analyzed different versions of HBV genome that encode HBx (National Center for Biotechnology Information, https://www.ncbi.nlm.nih.gov/), including AIB09443.1, AIB09391.1, and ABG23461.1, and found that these uploaded HBx protein versions all have a RFTS^75^AR (F, phenylalanine) region, a nonclassical binding domain for 14‐3‐3s. This area overlaps the MK2 phosphorylation sites (RXXpS/T) 43. Our work showing that the 14‐3‐3*ζ*‐HBx binding was not completely abolished when serine^31^ was mutated implied an existence of other potential binding sites of HBx for 14‐3‐3*ζ*. Based on the information mentioned above, we hypothesize RFTS^75^AR as a docking site for 14‐3‐3*ζ*. Further confirmatory experiments are being conducted by our group. Additionally, by analyzing the expression of 14‐3‐3*ζ* in clinic specimens, we found a higher expression of 14‐3‐3*ζ* in metastatic PVTT samples. These data corresponding to previous studies 9, 10, 30 suggested that the elevated 14‐3‐3*ζ* may be correlated with HCC development and metastasis. Although HBx positively correlated with 14‐3‐3*ζ* to be overexpressed in primary and metastatic tumor tissues, the *r* value is relatively low. As the correlation analysis between 14‐3‐3*ζ* and HBx expression in larger scale will enhance the importance of our work, more clinical specimens will be collected.

In summary, our work demonstrates that 14‐3‐3*ζ* binds to HBx in HBV‐infected HCC cells at least by docking to the RPLphosphoS^31^GP region of HBx. 14‐3‐3*ζ*‐slienced cells have decreased HBx expression and acquire a less malignant phenotype. These data shed lights on the role of 14‐3‐3*ζ* in HBV‐related hepatocarcinogenesis.

## Conflict of interest

No conflict of interest to declare.
